# Application of Stereolithography Based 3D Printing Technology in Investment Casting

**DOI:** 10.3390/mi11100946

**Published:** 2020-10-19

**Authors:** Muslim Mukhtarkhanov, Asma Perveen, Didier Talamona

**Affiliations:** School of Engineering and Digital Sciences, Department of Mechanical and Aerospace Engineering, Nazarbayev University, Nursultan 010000, Kazakhstan; muslim.mukhtarkhanov@nu.edu.kz (M.M.); asma.perveen@nu.edu.kz (A.P.)

**Keywords:** Stereolithography (SLA), investment casting, rapid casting, pattern, photopolymers

## Abstract

Advanced methods for manufacturing high quality parts should be used to ensure the production of competitive products for the world market. Investment casting (IC) is a process where a wax pattern is used as a sacrificial pattern to manufacture high precision casting of solid metal parts. Rapid casting is in turn, a technique that eases the IC process by combining additive manufacturing (AM) technologies with IC. The use of AM technologies to create patterns for new industrial products is a unique opportunity to develop cost-effective methods for producing investment casting parts in a timely manner. Particularly, stereolithography (SLA) based AM is of interest due to its high dimensional accuracy and the smooth surface quality of the printed parts. From the first appearance of commercially available SLA printers in the market, it took a few decades until desktop SLA printers became available to consumers at a reasonable price. Therefore, the aim of this review paper is to analyze the state-of-the-art and applicability of SLA based 3D printing technology in IC manufacturing, as SLA based AM technologies have been gaining enormous popularity in recent times. Other AM techniques in IC are also reviewed for comparison. Moreover, the SLA process parameters, material properties, and current issues are discussed.

## 1. Introduction

**Figure 1:**
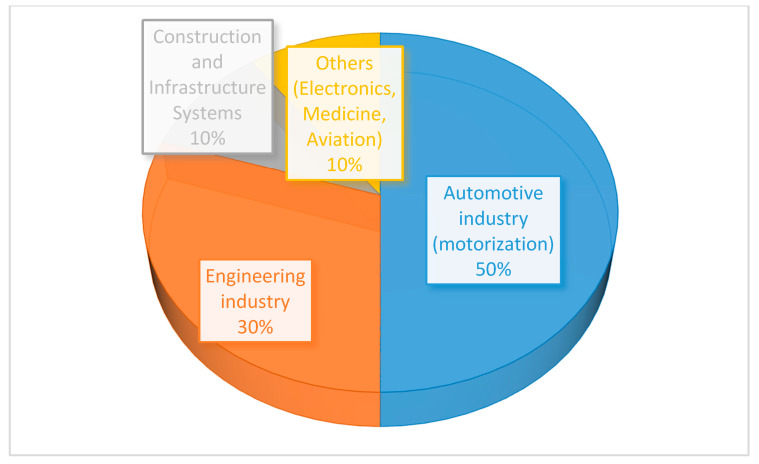
Main markets served by the foundry industry [1].

For thousands of years metal casting has been recognized as one of the main techniques for producing metal goods. Even today, it remains widely used by the industry. Holtzer et al. [1] indicated the constantly growing share of the foundry industry as a means of production for metal products. Despite continuous development of other production technologies, the foundry industry remains a significant and constant element of world economies. In regard to materials nomenclature, gray iron has a significant fraction of production volume. For example, in 2010 the global production in the foundry industry showed a 44 million ton production of grey iron, whereas the production of nonferrous metals showed 15 million tones, and steel 10 million tones. The market share of produced metals is shown in Figure 1. From the diagram it can be seen that the automotive, general engineering, and construction industries are the main market players. According to Holtzer’s study, the highest priorities for further development are modeling, prototyping, and production. Therefore, the advances in such areas such as rapid prototyping (RP) and rapid casting (RC) are of particular importance to ensure competitiveness of the foundry industries.

The process of casting is relatively simple and consists of the following steps: first the metal is melted, and then poured into prepared molds; after metal is solidified and cooled, the mold is destroyed, revealing the casted part. If the part has surface defects, it undergoes further machining to finish the process. Along with other casting methods such as die casting and sand casting, investment casting (IC) is regarded as an indispensable technology for casting metal products that have intricate shapes with high dimensional accuracy and superior surface finish [2]. On the contrary, sand casting allows the casting of larger objects and offers easier design changes. However, it is difficult in sand casting to achieve dimensional tolerances, shape complexities, low thickness walls, and surface finish equal to IC. To eliminate surface defects of sand casted parts, secondary machining processes are needed, which require additional labor and time. As far as die casting is concerned, it is a better option for manufacturing high or extremely high-volume products. The highly automated production pattern significantly reduces the cycle time for die casting. While both IC and die casting produce similar feature products, IC is capable of casting ferrous and non-ferrous metals alike. As die casting uses ferrous dies, it is mostly suited for casting non-ferrous alloys, with low melting points.

**Figure 2:**
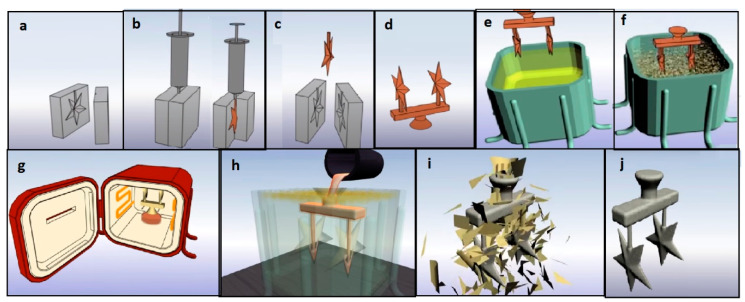
Schematic illustration of the investment casting (IC) process: (**a**) Metal mold prepared for wax injection; (**b**) Wax pattern making by injection; (**c**) Wax ejected from the mold; (**d**) Wax patterns assembled in a tree; (**e**) Coating with ceramic slurry; (**f**) Stucco coating; (**g**) Dewaxing through heating; (**h**) Metal pouring; (**i**) Destroying ceramic shell; (**j**) Casted part ready to be cut off from the assembly.

The IC process utilizes a wax pattern to create a ceramic shell that plays the role of the mold. The wax pattern is almost identical to the part that needs to be created considering the thermal shrinkage of the metal. Then, a ceramic slurry is applied on the surface of the pattern to generate the inner walls of the mold. The IC process sequence is shown in Figure 2. Depending on the production volume and other requirements, engineers exploit numerous tools for performing casting. However, what is common for all types of casting is the difficulty of tooling [3]. The most difficult part in the case of IC is the production of the mold for the wax pattern itself, which is done through manufacturing of a metal die. The metal die is injected with wax for pattern preparation. As the technology is fast moving forward, additive manufacturing (AM) may ease and replace the process of manufacturing the wax pattern to a great extent. AM is the process of manufacturing objects that applies a layer by layer approach with no additional tools. In this method, a CAD file of the object is created first. Then, the file is converted to STL format to accommodate the layer wise building process. The manufacturing itself is performed by using a 3D printer, that deposits layers of plastic, metal, or any other suitable material in a repeated manner so that the cross sections of the object build up on top of each other. Although, there are numerous of different AM machines available on the market, all of them share a similar manufacturing concept of building parts layer by layer.

Currently there is an increase in the number of AM technological solutions available; this is due to the massive interest in the technology itself, as the availability of these modern technologies and materials make it possible to design, manufacture, and test new parts rapidly. With decreasing cost, consumer grade printers can even be used at home. Additionally, AM technology has affected many areas of human activity such as culture, manufacturing, and even health care, for example. Successful application of AM resulted in making metal implantable devices [4], parts for aircraft production [5], and dental prostheses [6]. One of the significant roles of additive technologies is in shipbuilding: hydrodynamic testing of accurate models of ships, of any complexity, in the laboratory allows obtaining reliable data on the behavior of ships in conditions as close as possible to real [7].

**Figure 3:**
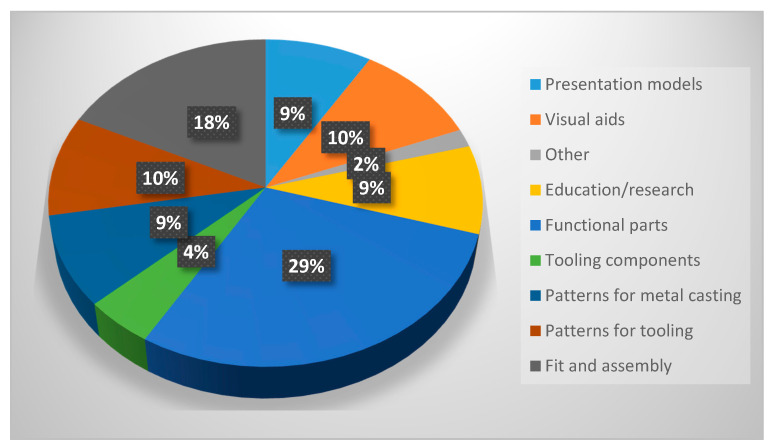
Most common applications for additive manufacturing (AM) [11].

Wohlers, a consulting agency, in its industry annual worldwide report (2014) [8] indicated an exponential increase in the use of AM in the production of finished, functional products. This shows that industry worldwide is gradually moving towards the new production technologies, and AM specifically. From Wohler’s report of 2016 [9], a pie chart of most common AM applications is shown in Figure 3. As can be seen, almost 30% of all AM technologies are in the areas of patterns for metal casting (9%), patterns for metal tooling (9.9%), and tooling components (4.7%). As for research publications concerning stereolithography (SLA), Ferry’s report (2012) [10] showed that only 35 out of 140 publications were about the technique itself, the rest being in the area of applications.

**Figure 4:**
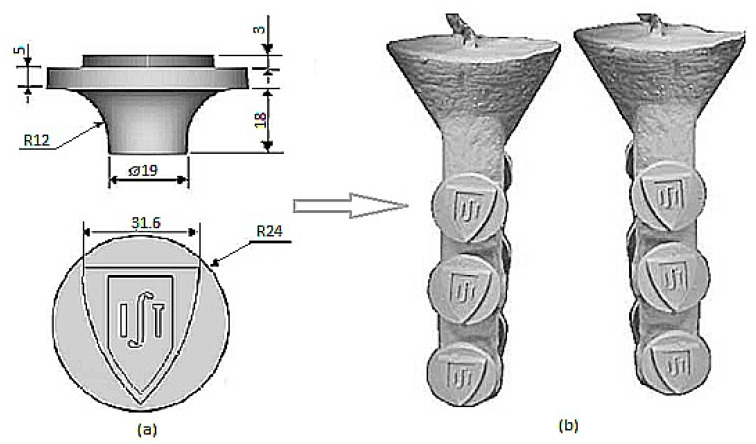
Cad model (**a**) and the pattern invested with ceramic shell (**b**) [13].

The interest in using AM for IC industry started shortly after the first AM technology was developed. One of the publications on using SLA for RC was done by Paul F. Jacobs as early as 1992 [12]. In particular, several case studies were presented where SLA parts served as wax substitutes for low run production. One case showed that casting of aluminum alloys with quality levels of grade D and class 3 could be achieved, which are typical engineering requirements. Figure 4 shows a CAD model of the pattern and the printed patterns prepared for casting [13].

Five main advantages of using SLA were highlighted:

- During the design cycle engineers can make changes to the model without compromising manufacturing lead time;
- Prototypes are useful for verification and assembly design; the case study showed that every fourth design change was due to defects revealed by the prototype model;
- The assembly of RP models served design optimization that helped to ignore unnecessary engineering change notices. As a result, cycle time can be reduced greatly;
- Elimination of the cost requalification of casting. As casting is integrated into the first production phase, there is no need to perform mechanical testing of initially casted parts. Therefore, the new approach has less expenditures;
- Reduction of scrap, due to shortening of machining operation.

Table 1 shows the comparison between SLA, traditional wax pattern, and machining from stock material methods. It is clearly shown that incorporating SLA into the casting process significantly lowers both the cost and lead time.

One of the earliest example of when SLA was used for rapid tooling (RT) demonstrated a cost reduction of more than 80%, as reported by Mueller [14]. The SLA was used to create patterns for silicon molds. It took two weeks to complete the task, whereas the estimated time for traditional tooling would have been seven weeks. Another example provided by Mueller [14] was the successful use of the QuickCast method, where water-swirlers were prototyped in less than a week, with reduced cost. Raja et al. investigated industrial applications of different AM technologies, including SLA, to test the feasibility of rapidly manufacturing high value aerospace parts in 2006 [15]. Their study concluded that three main benefits could be obtained by applying rapid manufacturing innovations in the industry: production lead time decrease by 30% to 70%; a 45% economy on non-recurring product introduction costs; a manufacturing cost reduction by 30% to 35% for low volume production parts.

Despite considerable developments in the 1990s and 2000s, the application of AM did not reach beyond the industrial level at that point of time. It was not until 2012 when the first desktop printers under $5000 arrived on the market, bringing enormous success to the manufacturers. From 2012 onward, AM technology started to reach market shares far beyond industrial businesses. The highly competitive market over the last decade brought tremendous technological innovations to the consumers. Nowadays, desktop 3D printer technology, which meets consumers’ expectations, has become an important part of the AM market. Therefore, it is important to analyze the current state-of-the-art of AM in conjunction with IC. Thus, the aim of this paper is to investigate whether SLA based AM technology is an effective tool for the IC industry. To do so, a comparison of SLA and other AM technologies that are also used in IC will be made. This analysis contains information on process parameters as well as the properties of SLA printed parts and the polymers used. Special attention was given to the dimensional accuracy of SLA printed parts, as it is one of the most important requirements for IC. The last part of the paper discusses current issues and limitations related to SLA applications in IC.

## 2. Role of Additive Manufacturing in IC

Ideally, direct manufacturing of metal parts using AM would be a perfect solution for manufacturers. However, metal AM for most industrial sectors is still at a stage of research and development, with limited industrial applications when compared to the full potential of metal AM. The high cost of machines and consumables, long printing time, and inferior quality of printed parts are some of the factors that hinder the widespread application of metal 3D printing [16]. Nevertheless, continuous development of metal AM makes it popular in aerospace, biomedicine, and automotive industries [17]. The two most popular metal printing technologies are powder bed fusion and direct energy deposition. The powder bed fusion method is similar to SLA in that the laser scans metal powder at the specified CAD file locations. Although the surface roughness and dimensional accuracy of metal AM is comparable to precision casting, micro pores, cracks, and residual stresses are present in printed parts [18]. Additionally, both technologies require post processing steps. For the SLA based IC, both printed and casted parts are subjected to post processing. As for printed part, the steps include support removal, and rinsing the part in alcohol for curing completion. Post processing of the casted part requires removal of feeders, heat treatment, and machining if needed. In the case of metal 3D printing, the post processing is limited to part removal from the printing platform, heat treatment, and machining. Nonetheless, both technologies are great tools for manufacturing highly customized, complex shaped products with low quantity.

There are different AM technologies other than SLA that have been used in the IC industry [19,20,21]. They include fused deposition modeling (FDM), selective laser sintering, laminated object manufacturing (LOM), etc. These techniques can be regarded as alternatives to SLA methods. Therefore, it is imperative to look at the performances of these technologies in IC to identify their pros and cons in comparison with SLA. These methods will be described hereafter in terms of their technology and performance in rapid IC as a direct method of pattern fabrication.

### 2.1. Fused Deposition Modeling

Fused deposition modeling (FDM) is perhaps the main competitor of SLA in the market. The printing mechanism of FDM is accomplished by the movement of a printer head that heats the thermoplastic filament to reach the melting temperature, and deposits it on the platform in a layer by layer fashion [22]. FDM is an extrusion based process that creates a structure from the bottom up. The most common materials used are polymers such as Acrylonitrile butadiene styrene (ABS) and Polylactic Acid (PLA) [23]. When FDM is used for IC it is assumed that ABS is superior to PLA, due to its better surface finish [24]. FDM can print both wax and non-wax patterns [21]. However, sacrificial non-wax patterns are superior in terms of mechanical properties and dimensional accuracy [19]. Moreover, it was reported that the wax patterns were even unable to carry their own weight [25,26]. Similar to SLA, early use of FDM non-wax pattern was associated with shell cracking issues. Recent developments in materials science have opened new opportunities for FDM in the area of IC. In particular, the emergence of the new Polycast polymer, which is popular for FDM in the foundry industry [27]. The obvious advantages of using FDM are the low cost of consumables [28] and machines compared to SLA, the wide range of machines, and easiness of pattern preparation [29]. The disadvantages of FDM are the low dimensional accuracy (±127 µm) as compared to SLA (±50 µm) [21], rough surface [30], and longer printing time, if high accuracy is aimed at. High surface roughness (*Ra*), being the major drawback of FDM technology, has been one of the major challenges for the AM industry. It has been found that process parameter optimization can solve the problem partially. For example, layer thickness is the most important parameter to ensure better surface quality, according to Jaisingh et al. [31]. However, the layer thickness has a certain upper and lower limit, thus, it is impossible to reach infinitesimally small layer thickness, to achieve the optimal value of *Ra*. On the other hand, post-process surface treatments can be utilized to reach desired outcomes. For instance, Jasgurpreed et al. [32] have suggested a chemical finishing process that can be applied to the FDM printed implant surface. The implant’s surface was exposed to acetone vapor for an optimal duration, and the *Ra* reached as low as a 27.2 nm value.

### 2.2. Selective Laser Sintering

During selective laser sintering, tiny particles of plastic, ceramic, metal, or glass are fused together using heat from a high-power laser to form a solid, three-dimensional object [22]. One of the earliest attempts at employing selective laser sintering (SLS) for printing patterns was made in 2003 by Liu et al. [33]. A polystyrene polymer powder was used as a raw material to create an impeller with an intricate shape. The rest of the processes were similar to those of traditional IC, except that vacuum casting was used for aluminum metal. The main difficulties of the process included dimensional inaccuracies and pattern burn out issues caused during the printing and casting. A completely different approach was utilized by Dotchev et al. in 2007 [34]. A two-stage process that consisted of: stage 1, fabrication of a green part using new CastForm polystyrene, and stage 2, infiltration by wax. This process is considered as one of the fastest and most inexpensive methods. However, the authors reported that during infiltration and cleaning stages the pattern was prone to breakage and distortion. Other disadvantages of the SLS technique include: the need for high ambient temperature and slow cooling rate; inferior quality of reused powder; incapability of using only a portion of the printing bed.

### 2.3. 3D Printing (3DP)

The 3D Printing (3DP) uses a so-called inkjet technology, which is similar to SLS with the difference that instead of sintering the powder with a laser, the powder bed is bounded together by droplets from a print head at specified locations [35]. The process repeats itself for each new layer of powder that covers the whole print bed. Many studies have been carried out for identifying the feasibility of using 3DP for rapid casting [36,37,38,39]. A prominent one is the study done by Gill et al. [36]. The method of pattern casting was also similar to what has been practiced in SLS techniques described earlier. The pattern was first printed using expandable starch and plaster-based powder. Further infiltration was done by immersing the patterns in liquid acrylate infiltration material for 10 min. Subsequent curing at +100 °C temperature finished pattern preparation. Additionally, extra printed green patterns were wax infiltrated for comparison. The experiments resulted in castings aluminum parts, most of which had dimensional accuracies accepted by foundry requirements for light alloys. The inkjet technology is regarded as the fastest and most cost-effective RIC among other AM techniques. However, it is also considered as the least accurate one [30]. An inkjet technology that offers much better accuracy and high quality surface finish comparable to SLA is called PolyJet. In PolyJet AM technology, droplets of photo-curable resin fall on the printing bed, and are subsequently scanned with ultraviolet light. The next layer of ink is applied to the previously cured line, and the process repeats until the object is built [40]. In a sense, PolyJet is similar to SLA technology. In contrast to the point by point laser scanning method, PolyJet supplies polymer droplets in a linear fashion, which increases printing speed significantly. Another noticeable advantage of Polyjet is the small layer thickness that can produce parts with high resolution and fine detail. However, the key characteristic of PolyJet AM, which makes it stand out from all the other technologies, is the vast variety of materials that can be produced with it. Flexible, rigid, and castable parts are all possible to print. Despite all the benefits that PolyJet has, the prohibitively high cost of machines and materials make it unpopular among AM technologies. Moreover, desktop machine configurations cannot be found in the market. The feasibility of PolyJet application in Direct RT was analyzed by Razvan et al. in 2017 [41]. According to the study, the mold prepared by PolyJet was suitable for the reaction injection molding of 300–500 plastic products.

### 2.4. Model Maker II

The Model Maker II (MMII) technique also uses inkjet technology; however, several jets are used. Two inkjets serve different purposes: one for building the pattern, and the other for making wax supports. The supports can be easily removed by applying solvents. What makes the multi-jet method special is the presence of additional machining, which is milling. The milling stage follows each layer to ensure uniform thickness. Although the extra stage greatly increases building time, it helps to generate surface finish with exceptional accuracy. That is why, it has been reported that MMII patterns have better accuracy compared to FDM [30]. Other advantages of the drop on drop technique include dense microstructure, lack of porosity, low melting temperature, and absence of shell cracking. According to Munish et al. [30], the application of MMII is limited to the small production of models having complex shapes.

### 2.5. Laminated Object Manufacturing

Laminated object manufacturing (LOM) employs a completely different technique of AM. During the process a sheet of paper, plastic, or metal covers the printing platform [42]. The next sheet is placed upon the previously laid paper. A heated roller presses the layers and melts the adhesive coating of the paper [22]. As a result, the layers are bonded to each other. Between the successive placement of sheets, a laser or knife cuts the outer perimeter of a predesigned cross section. The excess paper is used as supports and they are removed afterwards [43]. To ease the process of removal, the excess paper is cut into cubes. The process is notorious for generating smoke, which is caused by paper burning. To prevent paper swelling and delamination, the pattern undergoes post processing in the form of coating. Despite the fact that patterns lack high accuracy and surface quality, the technique is widely used for investment and sand casting objects with no thin walls [44]. The obvious advantage of the LOM are high speed and low cost. Table 2 summarizes the pros and cons of different AM technologies used in conjunction with IC.

## 3. SLA Working Principle

**Figure 5:**
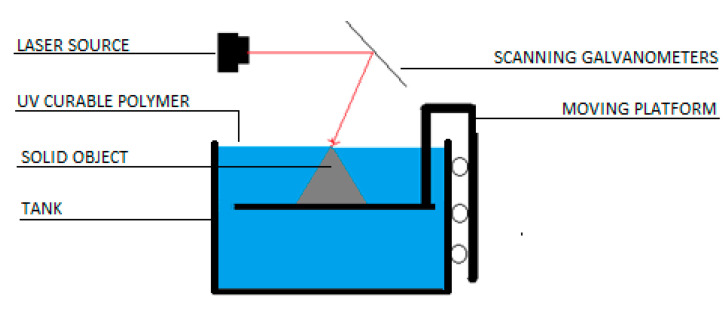
Schematics of SLA.

SLA is an additive manufacturing technology known as vat photopolymerization [11]. The machine uses a light source, a UV laser or projector, to cure liquid resin into hardened plastic. The main core components of machines are the light source, the build platform, and the resin tank (see Figure 5). The whole process of product development uses steps, which are all common to AM:

- CAD model generation on any suitable software;
- STL file generation (3D meshes of triangular elements);
- SLI file generation (model slicing);
- Manufacturing layer by layer using SLA printer;
- Post processing (removing built supports, washing and curing).

**Figure 6:**
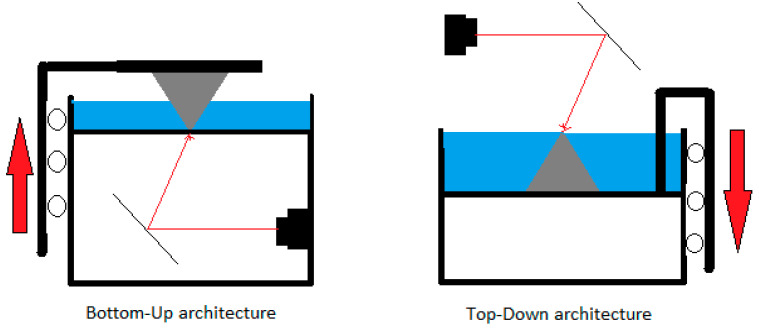
Schematics of different machine configurations.

The understanding of the effect of process parameters on the parts is essential for successful product design in any manufacturing industry. Although modern 3D printers come with already adjusted “default” process parameters, it is also important to understand the basic working mechanisms of SLA printers. The machine consists of a vat that contains liquid polymer, a platform upon which the object is built, a laser source, and a system of dynamic mirrors for guiding the laser beam. In some recent desktop configurations, the mirror is in a fixed position, and so-called galvanometers act as laser guiding devices, in x and y coordinates. The laser scans the surface of the liquid polymer in a point by point fashion. Once a layer is scanned, the surface needs to be covered with thin film of polymer again for consequent scanning. Here, it is important to understand that the main time consuming process is not the scanning, but the recoating [11]. And this is done by moving the platform up/down by a one-layer thickness. Figure 6 shows schematics of two machine configurations where the platform moves up (Bottom-Up) or down (Top-Down) to accomplish recoating. After recoating, it is important to swipe the surface of the polymer with a special blade, or wait for some small period, before the next scanning cycle occurs. The waiting periods are essential for several reasons. First, if the polymer has a high viscosity, the newly applied thin film of polymer develops surface tension that gives a convex shape to the layer [12]. Second, it was shown that a short waiting time decreases the curing time, therefore, weakening the interlayer bonding. The main disadvantage is that the extended waiting time significantly lengthens the overall processing time. To eliminate these issues, either a low viscosity photopolymer or swiping blade movement should be used. A combination of the mentioned low viscosity, with a swiping blade, was reported to bring about increased printing speed and interlayer strength.

**Figure 7:**
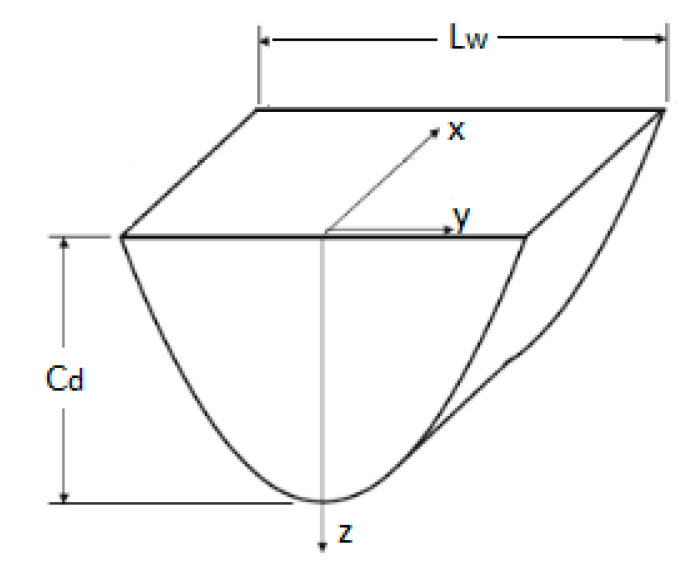
A cured line of a polymer during the SLA process.

During printing, the layer thickness is constrained by the wavelength of the laser and its spectral distribution that limit light penetration depths [49]. The curing depth (see Figure 7) can be calculated using Equation (1):

(1) $$C_{d}=D_{p}\ln\left(\frac{E_{max}}{E_{c}}\right)$$

where *D_p_* is penetration depth at which the beam intensity is reduced to 1/e of its surface value; *E_max_* is the maximum exposure energy on the resin surface; *E_c_* is the critical energy required for the transition of the resin from the liquid phase to the solid phase.

The maximum exposure energy on the resin surface can be adjusted to control the curing depth, and is a function of laser power (*P*) within the vat of the stereolithography apparatus, the beam radius (*w_O_*), and the scanning speed (*v_S_*). The relation given in [50] is Equation (2):

(2) $$E_{max}=\sqrt{\frac{2}{\pi }\;}\frac{P}{w_{O}v_{S}}$$

The energy at any point within the polymer thickness is given by Equation (3):

(3) $$E\left(x,y,z\right)=E\left(x,y,0\right)\exp\left(\frac{-z}{D_{p}}\right)$$

As for energy *E* at a given position x on the polymer surface is given by Equation (4):

(4) $$E\left(x\right)=E_{max}e\mathrm{xp}\left(\frac{-x^{2}}{w_{O}^{2}}\right)$$

The width of polymerized region can be defined as follows [12] Equation (5):

(5) $$L_{w}=B\sqrt{\frac{C_{d}}{2D_{P}}\;}$$

here *B* is the laser spot diameter.

If the laser spot diameter is of few micrometers, the SLA technology becomes a great tool to manufacture microparts, where layer thickness used decreases down to 1–10 µm [51]. In that case, SLA technology finds its application in so-called micro AM. The successful implementation of micro-stereolithography resulted in fabrication of photonic crystals [52], a biomedical micro device [53], rapid prototyping parts [54], and a temperature resistant hydrogel [55].

**Figure 8:**
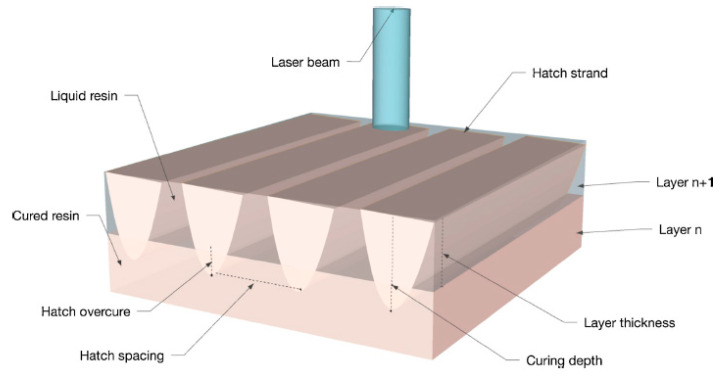
A section of a part generated by laser scanning [57].

In 2001 Lee et al. [56] studied the effects of the input parameters on the dimensional accuracy of SLA produced parts by applying a neural network approach. Their study reported that the layer thickness, hatch overcure, and hatch spacing are the most influential parameters in SLA process. The hatch overcure and hatch spacing are shown in Figure 8 [57]. As can be seen from Figure 8, during the printing process, the spaces between adjacent hatch strands may be left untraced. These untreated regions are controlled by hatch spacing. The liquid polymer within the hatch spacing can be cured during the post process treatment. If the hatch spacing is too small and the hatch overcure is large, the laser exposure will be increased, causing large volumetric shrinkage. On the contrary, too large hatch spacing leads to weak interlayer bonding. In addition, a small to non-hatch overcure positively affects the dimensional accuracy.

During the building process, the model may have inclined positions, creating challenges in terms of laser beam position. As a result, stair stepping effects emerge [58]. However, recent modifications in SLA machines, and optimization of process parameters, led to minimization of these surface defects. Once the building process is complete, several post-processing steps are needed to obtain the finished part. These include, 1. rinsing in water → 2. cleaning in ethyl alcohol → 3. removing supports (used for overhanging parts) → 4. post curing in a UV chamber. Like any other manufacturing process, the SLA technique does not produce defect free products. Defects might occur due to the improper choice of material or process parameters. Table 3 summarizes different types of defects, and their most probable origins.

The above mentioned defects are initially caused by material imperfections. However, recent advancements in polymer materials science have eliminated many issues to a great extent [50].

## 4. Description of the Properties of Printed Parts that Are Important in IC

**Figure 9:**
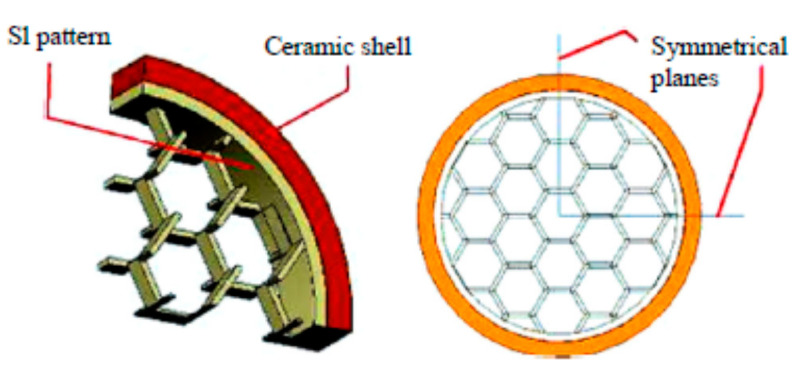
QuickCast 2 hexagonal build structure [70].

To produce high quality castings and ease IC process, it is desired for patterns to possess certain properties, such as high dimensional accuracy; low melting point for effortless burnout; high quality of surface finish; sufficient level of toughness and strength for comfortable handling and finishing; absence of residual ash after burnout; low thermal expansion for avoiding shell cracking. Among AM technologies, SLA is recognized as a highly accurate technique [30,56,68]. One of the unique advantage of the SLA method is the ability to apply a myriad of polymer types, including wax. However, wax patterns printed on SLA proved to be brittle and incapable of generating thin walls [21]. Moreover, a non-wax pattern is much superior in terms of accuracy. All these issues rendered SLA wax printing an unpopular technique. On contrary, employing non-wax patterns has the advantages of higher strength, durability, and toughness [69]. Another noticeable benefit of applying SLA in IC, is the smooth surface of printed parts, with surface roughness of 12.5 µm when applying epoxy [21]. As for the issue of ceramic shell cracking, SLA successfully uses QuickCast technology that creates a semi-hollow inner structure of pattern walls as shown in Figure 9 [70]. The role of the structure or infill is to minimize the negative effect of the high thermal expansion of polymers. The innovations that caused considerable development in SLA technology for the IC industry are summarized in Table 4.

Some of the pioneer research on the feasibility of using QuickCast for RC was done in 1995 by the European action on rapid prototyping [30]. The project aimed to evaluate the feasibility of using AM technologies in RC. To do so, QuickCast 1.0, FDM (wax pattern), LOM, SLS, and other techniques were compared. Most AM technologies employed a direct method with a lost pattern approach. Although the findings did not meet the researchers’ expectations in terms of accuracy, QuickCast was singled out as the most accurate method, and the most promising.

**Figure 10:**
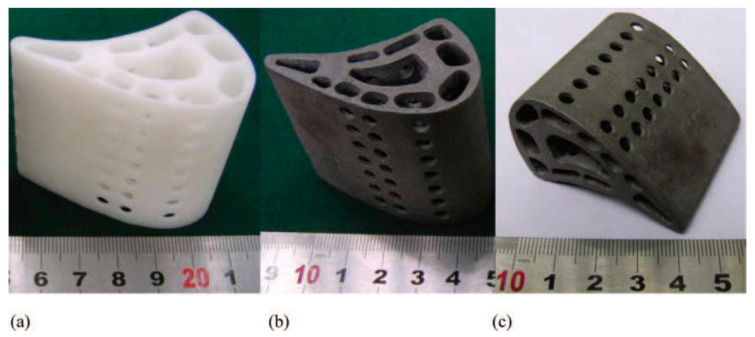
(**a**) The blade prototype; (**b**) the investment cast of the hollow turbine blade (front view); (**c**) the investment-cast of the hollow turbine blade (side view) [77].

Research conducted in 2008 by Wu et al. [77] provides an example of a novel RC method to manufacture a complex shaped hollow turbine blade. This novel method includes manufacturing with following steps: 1. CAD modeling → 2. Printing an SLA wax pattern → 3. Preparing ceramic slurry, and pouring on the pattern → 4. Gelcasting → 5. Vacuum drying → 6. Pyrolyzing → 7. Sintering → 8. Metal casting. The whole process took almost 25 days. According to estimates provided by the authors, a similar part would have taken over 170 days to manufacture using a traditional method. The method is regarded as innovative due to the inclusion of gelcasting for mold manufacturing. Aqueous colloidal silica was added to the ceramic slurry, and several benefits were observed. In particular, the new modified slurry composition allowed for gradual pyrolysis of the wax pattern, reducing thermal stress, as well as shell cracking. The obtained final product and printed model are shown in Figure 10 [77]. Although the research does not provide information on dimensional accuracy of the final product, this research opens new opportunities and avenues for further research and development in the direction of cost effectiveness and new tooling materials.

### 4.1. Dimensional Accuracy of SLA Printed Patterns

The dimensional accuracy of a pattern is one of the important requirements in IC, along with its surface roughness. The most suitable approach for assessing the accuracy level of fabricated parts is the evaluation of errors. To evaluate the suitability of the SLA technique, several researchers have investigated the dimensional accuracy of the printed and cast parts. Mathematical, process-related, and material related errors are defined as the primary sources of deviations [78]. One of the earliest reports published in this field belongs to Thomas H. Pang [72] who established the superiority of a new epoxy resin XB 5170 over a previously used acrylate based resin. According to Thomas H. Pang, the accuracy of the printed part was comparable to the parts machined by a CNC milling machine.

Rahmati et al. [79] in 2007 showed the applicability of SLA in producing silicon molds for wax patterns. The results showed that the phenomenon of error accumulation in a SLA pattern resulted from wax injection. According to the authors, a portion of deviation occurred during the SLA process, however, the exact ratio of that error was not identified.

G. Budzik et al. [80] tested SLA and 3DP in the rapid prototyping of a small turbocharger turbine impeller, having thin wall parts. The turbine impeller printed with SLA presented a higher accuracy as compared to the 3DP model, according to the study. Although the dimensional accuracies were not presented in numbers, the author recommends using the SLA method for producing the patterns suitable for silicon molds.

Similar research was conducted by Aamir et al. [81] in 2013, where a plastic mold was prepared using the SLA method to manufacture a turbine blade. Aamir et al. [81] successfully used SLA and FDM printers in RT. The aim of this study was to compare three materials that were suitable for mold production: polyurethane, room temperature vulcanized (RTV) silicon, and plaster. In parallel, two AM techniques were used to manufacture the patterns; FDM and SLA. Dimensional accuracies were measured and recorded for all the manufacturing stages. The findings were as follows:

- The concave and convex surfaces of the turbine blade had an average deviation of 160 µm and 220 µm, for SLA and FDM, respectively.
- As for the wax pattern, the majority of deviations were within a 500 µm tolerance limit.
- It was also suggested that SLA is a better option in terms of dimensional accuracy and surface roughness, in comparison to the FDM method for silicon mold making process.

High dimensional accuracy is extremely important in the field of biomedicine and dentistry. Alexey et al. [82], conducted a comprehensive study on the following printing parameters: building orientation, positioning, and curing, and their influence on the overall dimensional accuracy of 119 SLA printed parts. According to the findings, these parameters influence accuracy and the strength of parts. The findings are summarized in Table 5.

As can be seen from Table 5, the vertical printing orientation, z (90°), is the least accurate printing orientation. Other findings from the authors were that other parameters, such as sample position on the platform, also influence dimensional accuracy. For instance, samples printed close to the platform borders showed lower accuracy compared to the ones printed in the center.

The dimensional accuracy that SLA technology offers may allow the extension of SLA based IC technology in prosthetic dentistry. The quality of SLA based IC parts was investigated by Ami-Reza et al. in 2019 [83]. The study aimed to perform a statistical analysis, to compare the marginal fit of metal copings manufactured using different patterns. Copings are used in prosthetic dentistry, and are required to be made with high precision. A total of 30 patterns were produced by milling, SLA, and PolyJet. The results of the study showed that all three technologies were able to produce the metal copings within accepted tolerances, with PolyJet being the most reliable. The minimum and maximum values of the vertical marginal gap for the 10 tested SLA produced parts were found to be 42.25 and 127.5 µm, respectively.

Dimensional inaccuracy caused by the volumetric change during printing was studied by Islam et al. in 2016 [84]. The research aimed to compare dimensional accuracies of parts printed on a ProJet 3500 HDMax SLA printer and a powder binder printer (PBP). Table 6 contains data on the dimensional accuracies of objects printed by SLA.

The variations in dimensions were caused by the photo-polymerization process, according to the authors. The results of the comparison showed a higher dimensional accuracy and repeatability in the z direction for SLA. However, the average datum surface error was greater in SLA, by 68%, compared to PBP. The datum error occurrence was found to be due to the presence of convex curvature produced by the SLA process.

Relvas et al. (2012) [78] performed a comprehensive and exhaustive investigation on the dimensional accuracy of CNC (computer numerical control) and different AM techniques, including SLA. The authors used a systematic and rigorous approach for identifying dimensional accuracy and geometric precision of prototypes produced by numerous AM techniques and CNC. In order to achieve their goal, a benchmark was developed that contained 38 geometric shapes and 22 dimensional elements. Moreover, 12 points of the freeform surface were inspected for deviations. The study resulted in obtaining data for different types of dimensional and geometric deviations for CNC and four types of AM process: FDM, SLS, SLA, 3DP. Data for the SLA technique is shown in Table 7.

In comparison, the best result were obtained for CNC. The next best place was shared between SLA and SLS. The lowest accuracy level belonged to FDM and 3DP. The data in Table 6 for SLA clearly shows that most deviations were of geometric nature. As far as linear dimension deviation is concerned, even though half of the tested elements failed to meet ISO2768 requirement, the actual errors were close to the tolerance. Nevertheless, the SLA’s low performance, found by C. Relvas, contradicts the results of numerous other studies on the subject. The authors explained that one of the probable reason was that the other studies were conducted using different SLA machines [78].

### 4.2. Surface Roughness of SLA Printed Parts

The accuracy of patterns is also highly dependent on surface roughness (*Ra*). In many cases the Ra level of objects produced by AM technologies, including SLA, is unacceptable if precise parts are to be manufactured [85]. This is due to the layer-by-layer mode of production that generates a stair stepping effect on the part’s surface. In order to minimize or eliminate the undesired stair stepping features on the pattern surface, different measures have been developed. For instance, post processing treatments such as finishing can be used. In 1998, Williams et al. used a nontraditional finishing process, abrasive flow machining, to achieve *Ra* values of 1.27 - 3.53 µm. The obtained final *Ra* value depended on the magnitude of the original *Ra* value of the tested objects and the number of machining runs [86]. For example, the experiments showed that the first two runs of machining were the most resultative. Coating can also be used as an effective tool for post processing SLA printed parts. For example, a novel approach of applying polyethylene wax emulsion on the rough surfaces, through a ultra-high atomizing process, was proposed by Qiang et al. [85] in 2016. By applying a less than 0.1 mm thin layer of coating, it was possible to reduce the *Ra* value from 17 to 2.1 µm, for the SLA part printed with a manufacturing angle of 10°, according to the study. It was also shown that regardless of the manufacturing angle, coating results in more or less the same *Ra* value, which is around 2.5 µm.

Apart from post processing measures, it is possible to reduce *Ra* value to a certain degree by adjusting SLA processing parameters. The printing parameters that affect the surface roughness are shown in Table 8.

It should be noted that some processing parameters need to be optimized in combination with other parameters, such as resolution, laser exposure time, building direction, and the temperature inside the pool bath [89]. Thus, it creates an immense amount of difficulty to find an optimal combination of adjusted process parameters, since the amount of variables is not small. To overcome this problem, optimization techniques, such as the Taguchi method of optimization, should be used to adjust the process parameters. Unfortunately, the optimized process parameters may not work with the same level of efficiency for all types of resins or printing machines. For example, resin properties like viscosity and reactivity vary within different resins. Nevertheless, SLA technology offers a higher quality of surface finish in comparison with many AM technologies [21], and for manufacturing high precision parts, post process or process optimization treatments can be prescribed.

### 4.3. Mechanical Properties of SLA Printed Patterns

During handling, transportation, or even at investing stage, the wax patterns are subjected to forces that can damage them. That is why polymer patterns are a better choice compared to waxes. Especially, when thin walled or delicate parts need to be manufactured. The commercially available wax materials have tensile strengths and moduli of elasticity at around 10 and 200 MPa, respectively [90], whilst popular photo-curable epoxy resins have an average modulus of elasticity of 2200 MPa, and a tensile strength close to 50 MPa [91]. This striking difference between the mechanical properties of wax and SLA patterns clearly demonstrates the advantage of using polymer patterns.

## 5. Photopolymers and Issues in SLA Technology

The raw materials used in the SLA process are called photopolymers or photoresins. Their properties, both in the green and cured state, have a great influence on the quality of processing, and the properties of printed parts. In general curing of liquid resin is governed by the polymerization process, with monomers reacting with each other to form long chains and three-dimensional networks. From basic polymer chemistry, it is known that polymerization can be achieved by either chain-reaction or step-reaction polymerization. In the case of SLA, chain-reaction polymerization governs the process where free radicals act as building blocks between monomers. What is intrinsic to the polymerization process used in stereolithography is that ultraviolet (UV) light is used as an energy source to initiate photopolymerization [92]. The UV radiation is absorbed by a component called a photoinitiator [93], which in turn releases free radicals once activated. The subsequent step is crosslinking and/or polymerization, which turns liquid monomers into a solid polymer. Less energy consumption, reduced waste, fast curing, and lower reaction temperature are the main advantages of photopolymerization over conventional polymerization. In light-induced polymerization, the wavelength and spectral distribution limit light penetration depths, allowing the reaching of a few millimeters at maximum. This primary limitation turns out to be an advantage for the SLA method, where curing is achieved in a layer by layer manner. Photopolymers constitute the whole family of polymers subject to photopolymerization. It is estimated that close to half of all polymers on the AM materials market are photopolymers [11].

### 5.1. Free Radiacal and Cationic Systems

Free radical systems, based on acrylate and methacrylate monomers, were used as common stereolithographic polymeric resins in the infancy stage of SLA. In contrast, modern photopolymers are mainly based on radical and cationic systems [11].

Cationic photopolymerization was developed in the 1970s as an alternative to radical systems [72]. Due to several shortcomings intrinsic to radical photopolymerization, including high volume shrinkage and high viscosity, researchers found several compounds of onion salts that are thermally stable [11]. These salts, when exposed to UV light, decompose to form a mixture of cations, radical cations, and radical intermediates. The cationic mechanism uses epoxides and ether as the most important systems, among other constituents of the polymer. Epoxides are slow in reaction; therefore, they inhibit shrinkage and distortion. Another difference of cationic mechanisms is that scavenging of molecular oxygen is eliminated due to the presence of high active center concentrations. In turn, this allows spontaneous reaction continuation after the irradiation has ceased, which results in polymerization, even without the presence of light at room temperature [94]. The most common SLA epoxide monomers are diglycidyl ether derivatives of bisphenol A (DGEBA) [95], 3,4-epoxycyclohexylmethyl-3, 4-epoxycyclohexanecarboxylate (ECC), and epoxides of aliphatic alcohols, such as trimethyloyl propane. To integrate the advantages of radical and cationic systems, a hybrid system was developed, where the acrylate portion reacts significantly faster, while the epoxy portion undergoes a significant “dark cure”. Thus, hybrid systems, containing both radical and cationic monomers along with initiators, have become the standard mixtures today [93]. Table 9 presents some commercially available resins and photoinitiators, with their properties, used in SLA.

A two-stage curing reaction that consists of gelation and vitrification is used. During gelation, branching of molecules take place, thus increasing the viscosity of the medium. At this stage, the system cannot flow, and two phases coexist: a gel phase and a sol phase. Branching of molecules during gelation is possible only at a particular fractional conversion for each system, depending on the functionality, reactivity, and stoichiometry of the reactive species [50]. As for vitrification, the process is associated with rubber–glass transition. Vitrification is differentiated from gelation by reaction rate; here the reaction rate undergoes a significant decrease due to the increase of molecular weight and number of crosslinked parts. Another significant feature of the vitrification stage is the change in specific volume, resulting in stress generation. With the increase of curing time, a rise of both molecular weight and crosslinking density is observed. This in turn lowers the free volume, leading to an increase in density and elastic modulus. By contrast, free volume excess is formed with an increase of curing rate [11]. The parameters that affect curing kinetics include light intensity, temperature, and resin composition. To be precise, high light intensity, temperature, and concentration of photoinitiator cause rapid curing. Although rapid curing causes little volume change, poor mechanical properties can also be expected [50].

Monomers and photoinitiators, being the most important components of photopolymers, are not the only constituents of modern compounds. Numerous other additives serve different purposes, among them: flexibilizers, stabilizers, colorants, and many more. As polymer chemistry provides an enormous variety in materials, new compounds with enhanced properties emerge frequently in the market. Recent innovations in the field include new photoinitiators that are water soluble and visible-light-sensitive [100]. Regarding monomers, new developments mainly concern the enhancement of mechanical properties, such as toughness, flexibility, and the shape memory property. Despite major advances having occurred in the field, commercial photoinitiators and monomers still suffer from various limitations, according to Barner-Kowolik [101]. Moreover, the high cost of photopolymers still remains a limiting factor for widespread usage of SLA technology in IC [102]. As far as sustainability issues are concerned, there is a certain level of concern regarding the unknown effects of new materials on human health [103].

### 5.2. Non Polymer Materials Printed by SLA

**Figure 11:**
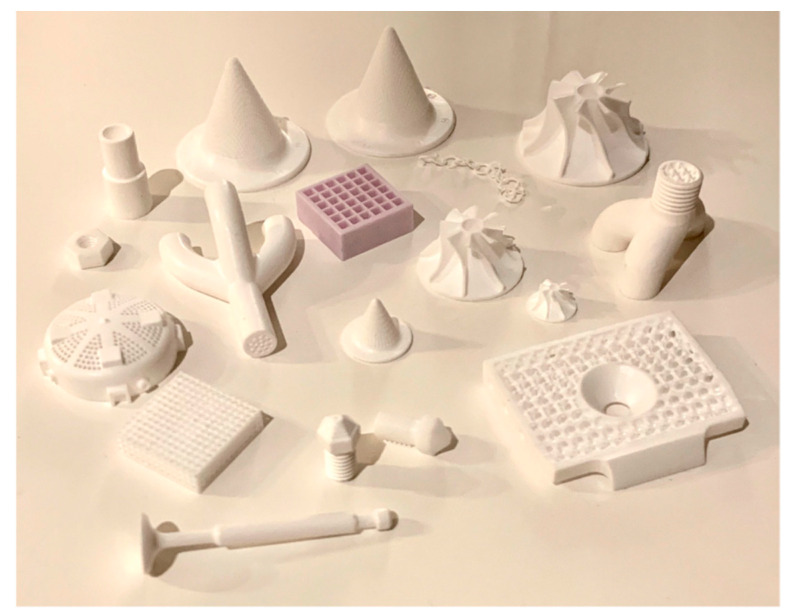
Example of objects made from ceramic resins; printed on CeraFab 7500 [57].

Stereolithography technique has not been restricted to using only polymeric material. For instance, there are numerous cases where SLA was employed to build metal and ceramic parts for IC [104]. Photopolymers are prone to having poor mechanical characteristics compared to metallic or ceramic parts. Joining metal/ceramic powder using photo-curable resin as a binder, has long been attempted by many researchers. It is reported that powders such as silica, silicon nitride, alumina, hydroxyapatite, lead zirconate titanate (PZT) oxides, aluminum, and copper have been successfully used [50]. Figure 11 shows an example of parts printed using alumina and zirconia [57].

Bartolo et al. [50] have listed a number of conditions needed when using ceramic/metal powder based SLA. The main ones are:

- parts should undergo debinding and sintering steps after manufacturing;
- the viscosity of the suspension should be low enough for proper recoating to take place;
- the suspension should have a sufficient amount of powder to ensure the satisfactory mechanical properties of the product that has gone through rebinding and sintering;
- the curing depth and resolution must be appropriate; the presence of powder particles interfere in absorption of beam light by the polymer. Thus, the curing depth decreases, leading to an increase of processing time.

Chartier et al. in 2002 [105] successfully manufactured aluminum sample impellers using SLA with *d*_50_ = 1.5 µm Al powder. Printed parts were sintered and the density of the final parts was reported to achieve 97% of the theoretical value, with flexural strength of 275 MPa. The research group have defined a number of important processing parameters and material properties suitable for successful production of parts that can be readily used in RP and IC. For example, the relation was found between powder concentration, scanning speed, and energy density of the light. According to the authors, an accuracy of ±0.5% could be obtained, provided the process parameters are chosen correctly, and the part has a size of a few centimeters. However, no information was provided regarding surface roughness and overall manufacturing duration.

The example of SLA printing of ceramics part was given by Weizhao et al. in 2010 [106]. The printed part had a final density equal to 62.45% of the theoretical measure, which is considered as insufficient. Nevertheless, the findings contain useful information on preparation of optimal photo-curable suspension, in terms of viscosity behavior and UV reactivity. Other research limitations were lack of information on the dimensional accuracy and surface roughness of the final part. Table 10 represents a list of selected research works dedicated to SLA of metal/ceramic parts.

RC of ceramic parts using SLA still remains at laboratory scale. This is due to the low performance of ceramic/metal SLA printed parts in terms of dimensional accuracy, and the overall mechanical properties of both green and sintered parts.

### 5.3. Ceramic Shell Cracking

One of the significant factors that limits the widespread application of SLA in IC is the issue of ceramic shell cracking. Although the QuickCast platform substantially contributed to solving this problem, IC of small parts with thin sections remains a challenge [15]. Moreover, QuickCast’s hexagonal build style can be improved or upgraded to a new architecture for more efficient performance during the burn out process [70,110]. For instance, in 2016 Gu et al. performed a topology optimization for the inner lattice structure. By applying thermo-mechanical finite element analysis (FEA), it was possible to generate a new inner lattice configuration, which was superior to the existing QuickCast 2.0 system for the given part geometry. The authors concluded that the proposed optimization method was universal and applicable to any type of pattern. Despite the lack of experimental confirmation, the study showed that there is no universal type of the inner lattice structure suitable for all types of part geometry. Other studies on the ceramic shell cracking problem are presented in Table 11.

One solution for overcoming the problem of shell cracking might be strengthening the ceramic shell [119,120,121]. For example, Jones et al. in 2003 [122] proposed a novel method of introducing organic fibers to the ceramic slurry. The authors concluded that adding nylon fibers with a diameter of 20 µm and a length of 1 mm can add strength to the shell, and as a result reduces the number of coatings. Although the research concerned a standard IC foundry, it opens the possibility of applying this technique for non-wax patterns. However, it should be noted that the method of strengthening ceramic shell is associated with certain limitations dictated by IC requirements. One of the requirements states that a high strength ceramic shell can lead to hot tearing of susceptible alloys. In addition, the mold material should have adequate permeability and thermal conductivity to ensure correct cooling [2]. Therefore, ceramic slurry modification should be tailored to particular casting type, and factors for consideration include material type, and the complexity and size of the casting.

Another approach for tackling the issue of ceramic shell cracking is in the area of polymer composition modification [123]. In 1989, Murphy et al. [124] proposed a SLA polymeric composition applicable to IC containing an inert low thermoplastic material. The role of the thermoplastic additive was to reduce the effect of expansion of the pattern upon heating. According to authors, the inert thermoplastic oligomer would locate in interstitial locations of the crosslinked polymer and soften the latter when heated. During the study, a 5 to 50 weight percentage of thermoplastic oligomer, with melting temperature below 100 °C, was added to the photopolymer to mark weight loss during heating up to 400 °C. The authors stated that weight loss of polymer at elevated temperatures was indicative of a polymer’s relaxation, due to the melting of thermoplastic component. However, the research lacks information on actual experiments on ceramic shells.

## 6. Current Research Trends and Challenges

The analysis of recently published articles revealed very few areas that concern SLA application in IC. It can be concluded that the main reason for that is economical. Although the industry is witnessing substantial development of AM technologies, the application of SLA in IC remains economically justified only for small quantity production. The current research trends include topology optimization, improving surface finish, and solving the ceramic shell cracking problem. The study identified probable aspects where further research and development might be needed:

- The majority of research publications on the topic are ten or more years old, thus, new research projects are needed, which might investigate new materials, technologies, and machines currently available.
- The research is scarce on the application of SLA in indirect tooling.
- Limited publications are available on the performance of new polymers suitable for IC;
- An important constraint to the incorporation of RC in foundries is still the high cost of AM machines, building materials, and consumables. Therefore, it is important to search for means of making the manufacturing process more cost effective.
- The ceramic shell cracking phenomenon remains an issue for parts with thin walls and complicated shapes. Few ways of solving this problem have been proposed by researchers. The two main directions of investigations are materials and process based approaches.
- Although the dimensional accuracy of SLA printed parts is higher compared to most AM technologies, it is still inferior to conventional CNC machining.
- Surface roughness is critical in manufacturing precise aerospace and medical products. Therefore, these fields require very low surface roughness values. Like many AM processes, SLA is prone to having a stair stepping effect. Improving the surface quality of SLA printed parts can be achieved through improving process parameters and advancements in materials science. Thus, deep knowledge is required to identify the major parameters that affect the surface roughness.
- The toxicity level of widely used photopolymers should be studied to evaluate the level of risk and perform a proper life cycle analysis of SLA based IC process.

## 7. Conclusions

The aim of this paper was to review the current status of stereolithography based AM technique in IC manufacturing. Based on the literature review, it can be concluded that there is solid evidence to consider SLA as a great tool for manufacturing both patterns and tooling, which are the main elements of IC industry. Started as rapid prototyping tool in the 1980s, SLA has extended its application area to include rapid IC and beyond. It has been shown that the unique properties of SLA printed parts, like high dimensional accuracy and good surface finish, led to its popularity in the aerospace, automobile, and biomedical industries. With the emergence of desktop printers in the market, a unique opportunity is provided for consumers, small business, entrepreneurship, and academia, who were previously unable to afford the high cost industrial size machines. This paper has also presented basic principles of how SLA works, along with information on photopolymers, which are the raw materials of SLA. The polymer industry, being highly innovative, creates additional incentive for researchers to use new polymers to minimize existing deficiencies involved with currently used materials. One problem that still needs to be overcome is the high thermal expansion of resins that sometimes causes ceramic shell cracking. Economists forecast a substantial growth of investments in additive manufacturing over the next few years. Despite controversial concerns on the sustainability of AM technology, it is believed that AM is likely to revolutionize existing manufacturing trends. This is due to technological progress, and the gradual switching of the market towards customized products. Thus, further investigation and improvements are needed to perfectly incorporate SLA based AM into IC. This is needed for the foundries to properly respond to future market demands [122].

## Figures and Tables

**Table 1:** Comparison of manufacturing cost and cycle time [12].

Features Compared	Casting with Stereolithography (SLA)	Traditional Wax Pattern	Machining from Stock Material
Cost, thousands of $	215	330	550
Cycle time, months	5	8	7
Length of time for incorporating design changes, months	2	1	1

**Table 2:** Qualities of patterns printed on different AM machines.

Type of AM	Advantages	Disadvantages	Source
SLA	High dimensional accuracy and fine detail; high quality surface finish; wide range of materials can be printed including wax and non-wax; relatively fast; low cost of desktop machines; transparent parts.	High cost of consumables; resin drainage of thin walled parts is a challenge; working with QuickCast technology requires experience; post-processing is required.	[15,21,30,45]
FDM	Both wax and non-wax patterns can be printed; strong non-wax parts can be handled easily; high printing speed; can print thin walls; large variety of machine configurations with comparatively low cost; low cost of consumables.	Poor surface finish; high melting temperature of Acrylonitrile butadiene styrene (ABS); to achieve high surface finish and high dimensional accuracy, many process parameters, layer thickness being the most prominent, need to be adjusted, which leads to long printing time.	[21,25,30,46]
SLS	Surface roughness close to SLA; high speed of printing; using polystyrene based Windform material allows casting of highly reactive alloys; easy post processing and removal of support structures when using True Form PM material.	High thermal expansion when using Duraform material; low dimensional accuracy with polystyrene material; incapability of using only a portion of printing bed; high cost of powder.	[21,30,33,34,47,48]
3DP	Low cost; very high speed of printing	Very low level of accuracy; poor surface finish.	[30,35,36]
Model Maker II	Can produce wax patterns; high quality of surface finish; no shell cracking.	Slow printing speed; fragility of wax material.	[21,30]
LOM	Low cost; high speed.	Low dimensional accuracy; inferior surface quality; more suitable for sand casting.	[25,44]

**Table 3:** Defects intrinsic to SLA printed parts.

Defect Type	Caused by
Shrinkage	Forming large molecules from small ones during the polymerization process; post-curing of the liquid resin trapped in the lattice structure [12]. Reaction related heat: ceasing of the initial photo-polymerization reaction causes temperature decrease [59].
Curl distortion	Curing process of each layer produces flexure of the layers previously solidified [56,60]. The part’s surface closest to the bottom of the platform curls most [61]. Layer thickness, hatch spacing, fill cure depth, and hatch over-cure [62].
Warping	Insufficient polymerization leading to relaxation, diffusion, and evaporation of low molecular weight components [50]. Instantaneous increase in temperature and subsequent release of heat to the surroundings [63].
Distortions Dimensional inaccuracy	Liberation of internal forces that have developed during the building process between the model and the platform [56] Resin’s photosensitivity characteristics and laser power [64]. Deviation of the laser beam when curing resin far from the platform center [65]. Hatch spacing, scanning speed and coefficient of the resin’s shrinkage compensation are the most influential parameters affecting dimensional accuracy [66]. Incorrect selection of part orientation [67].

**Table 4:** Historical development of SLA technology.

Year	Innovation
1986	3D Systems Company founder Chuck Hull receives his patent for the first SLA apparatus [71]
1988	3D Systems with its partner Ciba-Geigy introduced the first-generation acrylate resins. The resins had issues such as high viscosity, dimensional inconsistency, low strength, and brittleness [72]
1993	Commercialization of the first XB 5170 epoxy resin product. Despite the slowing down of photo-speed process, introducing epoxy/acrylate hybrids allowed lowering viscosity and improving both green and impact strengths [73,74]. Emergence of QuickCast 1.0 technology. The QuickCast innovation allows implementing a build style that makes inner the volume of pattern walls have a porous structure. Due to a considerable decrease in density of quasi-hollow parts, the inner part of pattern walls collapse before ceramic shell cracks [75]. As a result, the effect of polymer thermal expansion is almost eliminated [21]. Moreover, as less material is needed for printing hollow-like structures, material savings are another benefit of applying the QuickCast method.
2000	Through further development of QuickCast technology, new building styles have been introduced. For example, Figure 8 shows the hexagonal build style model of QuickCast 2.0. The superiority of QuickCast 2 over the previous version was in the reduction of shell stress by 2/3 [30].
2012	First desktop printers under $5000 arrived on the market.
2015	Introduction of a new continuous liquid interface production system that significantly increased the printing speed. The system has a new oxygen permeable vat bottom, which allows continuous polymerization of the resin and elimination of the recoating step [76].

**Table 5:** Mean values and SD for height, width, and length [82].

Mean Values, mm	3D Designed Value, mm	0° Build Orientation	45° Build Orientation	90° Build Orientation
Height (*x*)	5	4.5 (SD* = 0.11)	4.9 (SD = 0.02)	4.96 (SD = 0.01)
Wight (*y*)	5	5.11 (SD = 0.06)	4.82 (SD = 0.01)	4.95 (SD = 0.01)
Length (*z*)	30	29.94 (SD = 0.05)	29.89 (SD = 0.03)	29.25 (SD = 0.08)

SD*—Standard Deviation in mm.

**Table 6:** Dimensional errors of SLA printed parts [84].

Input Parameters	Unit	SLA				
		Height	Height	Hole Dia.	Outer Dia.	Outer Dia.
Design size	mm	10	60	30	46	126
Measured mean size	mm	10.367	59.995	29.859	45.768	125.394
Dimensional error	µm	367	−5	−141	−232	−606

**Table 7:** Results of tests on dimensional and geometrical accuracies of a SLA printed benchmark [78].

Type of Deviation	Total Number of Tested Elements	Number of Elements that Failed to Meet Tolerance Order of ISO2768-mk
Deviation of the linear dimensions	6	3
Deviation of radius, diameters, and angles	16	5
Geometric deviation of flatness and cylindricity	17	5
Geometric deviation of perpendicularity, coaxiality, parallelism, position, and angularity	21	20

**Table 8:** Processing parameters that influence surface roughness of SLA printed parts.

Parameter	Effect
Layer thickness or resolution	Finer layer thickness results in a low value of *Ra* [87]
Blade gap	Medium blade gap is desirable, 0.005 inch [88]
Hatch space	Medium or low is preferable [88]
Position on the platform	The closer to the center the better [88]

**Table 9:** Commercially available photopolymers and photoinitiators.

Polymer	Properties	System Type
Crystic 272 (isophthalic polyester) from Scott Bader [96]	Hardness, flexibility, thermal stability, and high resistance	Free radical
EC 130 LV from Camattini (epoxy) [96]	High elasticity module, good thermo and mechanical properties, low viscosity, and low shrinkage	Cationic
SL-100 DMX™ [71]	Durable, tough, and complex parts can be made with high accuracy	Cationic
Irgacure 651 from Ciba–Geigy (photoinitiator) [97]	High polymerization rate and high phosphorescence quantum yield	Radical
Esacure 1187 from Lamberty (photoinitiator) [98]	Thermally stable and has high toughness; low odor	Cationic
Irgacure 250 from Ciba–Geigy (photoinitiator) [99]	High reactivity, absence of hazardous products	Cationic

**Table 10:** Selected articles on SLA application in making metal/ceramic parts.

Year and Authors	Main Findings	Limitations
1996, Griffith, M. L., and Halloran, J. W. [107]	The first time a silica part was printed using SLA for IC. Al and silicon nitride were also manufactured as structural parts. The parts were of a box shape; layer thickness was 0.15 mm. Sintered at 1550 °C, the part reached full density and showed no trace of interlayer boundaries.	Very fine powder size around 0.5 µm is required for adequate dispersion to take place. Process parameters and suspension viscosity should be finely tuned to achieve optimal conditions. Due to high refractory index, silicon nitride powder showed very low curing depth of 10 µm for 0.2 solid fraction.
2005, Corcione, C. E., Greco, A., Montagna, F., Licciulli, A., and Maffezzoli, A. [108]	The ceramic molds were produced on a SLA250 printer. Both green and sintered objects were used for casting Al. Low difference in refraction index between resins and silica powders ensured good reactivity and cure depth, as compared to Al powder.	Sintered parts did not allow for multiple castings due to the presence of defects. Green molds need to be designed correctly to compensate for large isotropic shrinkage. Sintering process requires high temperature treatment up to 1200 °C. The information on dimensional accuracy and surface roughness were not provided.
2008, Bartolo, P. J., and Gaspar, J. [96]	60 wt% tungsten carbide or cobalt were used as metal powder. Low powder sizes resulted in both the decrease in light penetration and the overall fractional conversion.	No data on mechanical properties and dimensional accuracy of printed part were given. Low powder size resulted in low penetration depth, high viscosity, and low fractional conversion of the polymer.
2011, Bian, W., Li, D., Lian, Q., Zhang, W., Zhu, L., Li, X., and Jin, Z. [109]	SLA was used to fabricate novel porous implants with pre-set channels for blood vessel implantation. The green part from β-TCP powder did not change either its inner structure or shape after sintering. The compressive strength showed 23.54 MPa, which is close to natural cancellous bone.	Due to scattering of the laser beam by ceramic powder, shrinkage of porous media took place. As a result, a large difference between designed and achieved porosity occurred. The high elastic modulus of the printed part is a limiting factor for application in biomedicine.

**Table 11:** The effects of different parameters on ceramic shell cracking when using non-wax patterns.

Parameters	Main Findings
Temperature	Ceramic shell cracking occurs below the glass transition temperature Tg of the resin [111,112]. Ceramic shell cracking at 200–250 °C was observed when using a photosensitive pattern [113]. Flash firing is preferable to gradual temperature increase during pattern burnout [114].
Inner lattice geometry	Buckling temperature of the inner web links is affected by web geometry; having long web links is beneficial, as they buckle at temperatures lower than shell cracking temperature [115]. A honeycomb structure has different elastic modulus at different orientations due to anisotropic nature of the honeycomb lattice; this fact is important when considering large metal castings [116]. Topology optimization can be applied to generate an appropriate structure for the inner lattice [117].
Shell’s thickness and geometry	Thin shells and the presence of sharp corners have a negative effect on shell cracking [116,118]

## References

[1] Holtzer M., Dańko R., Żymankowska-Kumon S. (2012). Foundry industry–current state and future development. Metalurgija.

[2] Kalpakjian S., Schmid S.R. (2009). Manufacturing Engineering Technology.

[3] Ingole D.S., Kuthe A.M., Thakare S.B., Talankar A.S. (2009). Rapid prototyping–a technology transfer approach for development of rapid tooling. Rapid Prototyp. J..

[4] Youssef A., Hollister S.J., Dalton P.D. (2017). Additive manufacturing of polymer melts for implantable medical devices and scaffolds. Biofabrication.

[5] Liu R., Wang Z., Sparks T., Liou F., Newkirk J. (2017). Aerospace applications of laser additive manufacturing. Laser Additive Manufacturing.

[6] Javaid M., Haleem A. (2019). Current status and applications of additive manufacturing in dentistry: A literature-based review. J. Oral Biol. Craniofacial Res..

[7] Evans B. (2012). Practical 3D Printers: The Science and Art of 3D Printing.

[8] Wohlers T.T., Caffrey T. (2014). Wohlers Report 2014: 3D Printing and Additive Manufacturing State of the Industry Annual Worldwide Progress Report.

[9] Caffrey T., Ian Campbell T.W. (2016). Wohlers Report 2016: Additive Manufacturing and 3d Printing State of the Industry Annual Worldwide Progress Report.

[10] Melchels F.P.W. (2012). Celebrating three decades of stereolithography. Virtual Phys. Prototyp..

[11] Ligon S.C., Liska R., Stampfl J., Gurr M., Mülhaupt R. (2017). Polymers for 3D Printing and Customized Additive Manufacturing. Chem. Rev..

[12] Jacobs P.F. (1992). Rapid Prototyping & Manufacturing: Fundamentals of Stereolithography.

[13] Ferreira J.C., Mateus A. (2003). A numerical and experimental study of fracture in RP stereolithography patterns and ceramic shells for investment casting. J. Mater. Process. Technol..

[14] Mueller T. (1995). Stereolithography-based prototyping: Case histories of applications in product development. IEEE Technical Applications Conference and Workshops. Northcon/95. Conference Record.

[15] Raja V., Zhang S. (2006). Rapid and cost-effective manufacturing of high-integrity aerospace components Rapid and cost-effective manufacturing of high-integrity aerospace components. Int. J. Adv. Manuf. Technol..

[16] Debroy T., Mukherjee T., Milewski J.O., Elmer J.W., Ribic B., Blecher J.J., Zhang W. (2019). Scientific, technological and economic issues in metal printing and their solutions. Nat. Mater..

[17] Ngo T.D., Kashani A., Imbalzano G., Nguyen K.T.Q., Hui D. (2018). Additive manufacturing (3D printing): A review of materials, methods, applications and challenges. Compos. Part B Eng..

[18] Song B., Zhao X., Li S., Han C., Wei Q., Wen S., Liu J., Shi Y. (2015). Differences in microstructure and properties between selective laser melting and traditional manufacturing for fabrication of metal parts: A review. Front. Mech. Eng..

[19] Carneiro V.H., Rawson S.D., Puga H., Meireles J., Withers P.J. (2020). Additive Manufacturing Assisted Investment Casting: A Low-Cost Method to Fabricate Periodic Metallic Cellular Lattices. Addit. Manuf..

[20] Rooks B. (2002). Rapid tooling for casting prototypes. Assem. Autom..

[21] Cheah C.M., Chua C.K., Lee C.W., Feng C., Totong K. (2005). Rapid prototyping and tooling techniques: A review of applications for rapid investment casting. Int. J. Adv. Manuf. Technol..

[22] Mahindru D.V., Mahendru P., Mahindru V., Mahendru P. (2013). Review of Rapid Prototyping-Technology for the Future. Glob. J. Comput. Sci. Technol..

[23] Melnikova R., Ehrmann A., Finsterbusch K. (2014). 3D printing of textile-based structures by Fused Deposition Modelling (FDM) with different polymer materials. IOP Conference Series: Materials Science and Engineering.

[24] Hafsa M.N., Ibrahim M., Wahab M.S., Zahid M.S. (2014). Evaluation of FDM pattern with ABS and PLA material. Appl. Mech. Mater..

[25] Kumar P., Ahuja I.P.S., Singh R. (2012). Application of fusion deposition modelling for rapid investment casting—A review. Int. J. Mater. Eng. Innov..

[26] Cannell N., Sabau A.S. (2005). Predicting Pattern Tooling and Casting Dimensions for Investment Casting, Phase II..

[27] Note, Investment Casting with PolyCast ^TM^. https://polymaker.com/Downloads/Application_Note/PolyCast_Application_Note_V1.pdf.

[28] Choudhari C.M., Patil V.D. (2016). Product Development and its Comparative Analysis by SLA, SLS and FDM Rapid Prototyping Processes. IOP Conference Series: Materials Science and Engineering.

[29] Harun W.S.W., Safian S., Idris M.H. (2009). Evaluation of ABS patterns produced from FDM for investment casting process. WIT Trans. Eng. Sci..

[30] Chhabra M., Singh R. (2011). Rapid casting solutions: A review. Rapid Prototyp. J..

[31] Sheoran A.J., Kumar H. (2020). Fused Deposition modeling process parameters optimization and effect on mechanical properties and part quality: Review and reflection on present research. Mater. Today Proc..

[32] Chohan J.S., Singh R., Boparai K.S. (2020). Vapor smoothing process for surface finishing of FDM replicas. Mater. Today Proc..

[33] Hongjun L., Zitian F., Naiyu H., Xuanpu D. (2003). A note on rapid manufacturing process of metallic parts based on SLS plastic prototype. J. Mater. Process. Technol..

[34] Dotchev K., Soe S. (2006). Rapid manufacturing of patterns for investment casting: Improvement of quality and success rate. Rapid Prototyp. J..

[35] Zhang H., Zhu T., Cao S., Shu X., Sun B., Hu Y. 3DP System Development Based on Lnkjet Printer and its Experimental Research. *Mach. Des. Manuf.*
**2012**, *7*. http://en.cnki.com.cn/Article_en/CJFDTotal-JSYZ201207046.htm.

[36] Gill S.S., Kaplas M. (2011). Efficacy of powder-based three-dimensional printing (3DP) technologies for rapid casting of light alloys. Int. J. Adv. Manuf. Technol..

[37] Upadhyay M., Sivarupan T., El Mansori M. (2017). 3D printing for rapid sand casting—A review. J. Manuf. Process..

[38] Marwah O.M.F., Sharif S., Zainol M.A., Ibrahim M., Mohamad E.J. (2014). 3D printer patterns evaluation for direct investment casting. Appl. Mech. Mater..

[39] Seleznev M., Shulz B., Cornie J., Zhang S., Sachs E., Serdy J., Cima M. (2000). Novel Near-Net-Shape Tool-Less Method for Manufacturing of Cast Metal Matrix Composites: Three-Dimensional Printing (3DP) of Ceramic Preforms Combined with Investment Casting Technology. SAE Trans..

[40] Fischer F. (2015). FDM and Polyjet 3D printing. Pop. Plast. Packag..

[41] Udroiu R., Braga I.C. (2017). Polyjet technology applications for rapid tooling. MATEC Web of Conferences.

[42] Park J., Tari M.J., Hahn H.T. (2000). Characterization of the laminated object manufacturing (LOM) process. Rapid Prototyp. J..

[43] Hugo P. (2008). Suitability of Layer Manufacturing Technologies for Rapid Tooling Development in Investment Casting. Ph.D. Dissertation.

[44] Mueller B., Kochan D. (1999). Laminated object manufacturing for rapid tooling and patternmaking in foundry industry. Comput. Ind..

[45] Jacobs P.F. QuickCast 1.1 and Rapid Tooling. Proceedings of the 4th European Conference on Rapid Prototyping & Manufacturing.

[46] Jain P., Kuthe A.M. (2013). Feasibility study of manufacturing using rapid prototyping: FDM approach. Procedia Eng..

[47] Sood A.K. Use of Fused Deposition Modeling Process in Investment Precision Casting and Risk of Using Selective Laser Sintering Process. 2012; pp. 1–5. https://www.idc-online.com/technical_references/pdfs/mechanical_engineering/USE%20OF%20FUSED%20DEPOSITION.pdf.

[48] van de Crommert S., Seitz S., Esser K.K., McAlea K. (1997). Sand, die and investment cast parts via the SLS selective laser sintering process. Rapid Prototyping and Flexible Manufacturing.

[49] Gojzewski H., Guo Z., Grzelachowska W., Ridwan M.G., Hempenius M.A., Grijpma D.W., Vancso G.J. (2020). Layer-by-Layer Printing of Photopolymers in 3D: How Weak is the Interface?. ACS Appl. Mater. Interfaces.

[50] Bártolo P.J. (2011). Stereolithography: Materials, Processes and Applications.

[51] Vaezi M., Seitz H., Yang S. (2013). A review on 3D micro-additive manufacturing technologies. Int. J. Adv. Manuf. Technol..

[52] Shoji S., Smith N., Kawata S. (1999). Photofabrication of a photonic crystal using interference of a UV laser. Optical Engineering for Sensing and Nanotechnology (ICOSN’99).

[53] Ikuta K., Hasegawa T., Adachi T. (2001). The optimized SMA micro pump chip applicable to liquids and gases. Transducers’ 01 Eurosensors XV..

[54] Bertsch A., Jiguet S., Bernhard P., Renaud P. (2003). Microstereolithography: A Review.

[55] Han D., Lu Z., Chester S.A., Lee H. (2018). Micro 3D printing of a temperature-responsive hydrogel using projection micro-stereolithography. Sci. Rep..

[56] Lee S.H., Park W.S., Cho H.S., Zhang W., Leu M.-C. (2001). A neural network approach to the modelling and analysis of stereolithography processes. Proc. Inst. Mech. Eng. Part B J. Eng. Manuf..

[57] Zakeri S., Vippola M., Levänen E. (2020). A comprehensive review of the photopolymerization of ceramic resins used in stereolithography. Addit. Manuf..

[58] Pham D.T., Dimov S.S., Gault R.S. (1999). Part orientation in stereolithography. Int. J. Adv. Manuf. Technol..

[59] Narahara H., Tanaka F., Kishinami T., Igarashi S., Saito K. (1999). Reaction heat effects on initial linear shrinkage and deformation in stereolithography. Rapid Prototyp. J..

[60] Bernhard P., Hofmann M., Schulthess A., Steinmann B. (1994). Taking lithography to the third dimension. Chim. Int. J. Chem..

[61] Huang Y.-M., Lan H.-Y. (2006). Compensation of distortion in the bottom exposure of stereolithography process. Int. J. Adv. Manuf. Technol..

[62] Jayanthi S., Keefe M., Gargiulo E.P. Studies in Stereolithography: Influence of Process Parameters on Curl Distortion in Photopolymer Models. Proceedings of the 1994 International Solid Freeform Fabrication Symposium.

[63] Hull C.W., Spence S.T., Lewis C.W., Vinson W., Freed R.S., Smalley D.R. (1993). Stereolithographic Curl reducTion. U.S. Patent.

[64] Salmoria G.V., Ahrens C.H., Beal V.E., Pires A.T.N., Soldi V. (2009). Evaluation of post-curing and laser manufacturing parameters on the properties of SOMOS 7110 photosensitive resin used in stereolithography. Mater. Des..

[65] Fadel G.M., Kirschman C. (1996). Accuracy issues in CAD to RP translations. Rapid Prototyp. J..

[66] Xu G., Jin J., Luo S., Qiu R., Pan H. Research on Optimizing Build Parameters for Stereolithography Technology. Proceedings of the 2009 International Conference on Measuring Technology and Mechatronics Automation.

[67] Cheng W., Fuh J.Y.H., Nee A.Y.C., Wong Y.S., Loh H.T., Miyazawa T. (1995). Multi-objective optimization of part-building orientation in stereolithography. Rapid Prototyp. J..

[68] Yarlagadda P.K.D.V., Hock T.S. (2003). Statistical analysis on accuracy of wax patterns used in investment casting process. J. Mater. Process. Technol..

[69] Tewo R.K., Rutto H.L., Focke W., Seodigeng T., Koech L.K. (2019). Formulations, development and characterization techniques of investment casting patterns. Rev. Chem. Eng..

[70] Norouzi Y., Rahmati S., Hojjat Y. (2009). A Novel Lattice Structure for SL Investment Casting Patterns. Rapid Prototyp. J..

[71] Wohlers Associates Inc (2015). Wohler’s report 2015—3D Printing and Additive Manufacturing State of the Industry. Annual Worldwide Progress Report.

[72] Pang T.H. Stereolithography Epoxy Resin Development: Accuracy and Dimensional Stability. Proceedings of the 1993 International Solid Freeform Fabrication Symposium.

[73] Jacobs P. (1994). Stereolithography 1993: Epoxy resins, improved accuracy, and investment casting. Coupling Technol. Natl. Need.

[74] Queiroz D.A., Cunha L.G., Duarte J.L.P., Neves A.C.C., da Silva-Concílio L.R. (2011). Influence of the casting material on the dimensional accuracy of dental dies. Braz. Oral Res..

[75] Jacobs P. (1995). Rapid tooling. World Cl. Des. to Manuf..

[76] Tumbleston J.R., Shirvanyants D., Ermoshkin N., Janusziewicz R., Johnson A.R., Kelly D., Chen K., Pinschmidt R., Rolland J.P., Ermoshkin A. (2015). Continuous liquid interface production of 3D objects. Science.

[77] Wu H., Li D., Tang Y., Guo N., Cui F., Sun B. (2009). Rapid casting of hollow turbine blades using integral ceramic moulds. Proc. Inst. Mech. Eng. Part B J. Eng. Manuf..

[78] Relvas C., Ramos A., Completo A., Simões J.A. (2012). A systematic approach for an accuracy level using rapid prototyping technologies. Proc. Inst. Mech. Eng. Part B J. Eng. Manuf..

[79] Rahmati S., Akbari J., Barati E. (2007). Dimensional accuracy analysis of wax patterns created by RTV silicone rubber molding using the Taguchi approach. Rapid Prototyp. J..

[80] Budzik G. (2007). Properties of made by different methods of RP impeller foundry patterns. Arch. Foundry Eng..

[81] Iftikhar A., Khan M., Alam K., Imran Jaffery S.H., Ali L., Ayaz Y., Khan A. (2013). Turbine blade manufacturing through rapid tooling (RT) process and its quality inspection. Mater. Manuf. Process..

[82] Unkovskiy A., Bui P.H.B., Schille C., Geis-Gerstorfer J., Huettig F., Spintzyk S. (2018). Objects build orientation, positioning, and curing influence dimensional accuracy and flexural properties of stereolithographically printed resin. Dent. Mater..

[83] Khaledi A.-A., Farzin M., Akhlaghian M., Pardis S., Mir N. (2020). Evaluation of the marginal fit of metal copings fabricated by using 3 different CAD-CAM techniques: Milling, stereolithography, and 3D wax printer. J. Prosthet. Dent..

[84] Islam M.N., Gomer H., Sacks S. (2017). Comparison of dimensional accuracies of stereolithography and powder binder printing. Int. J. Adv. Manuf. Technol..

[85] Yang Q., Lu Z., Zhou J., Miao K., Li D. (2017). A novel method for improving surface finish of stereolithography apparatus. Int. J. Adv. Manuf. Technol..

[86] Williams R.E., Melton V.L. (1998). Abrasive flow finishing of stereolithography prototypes. Rapid Prototyp. J..

[87] Jacobs P.F. (1995). Stereolithography and other RP&M Technologies: From Rapid Prototyping to Rapid Tooling.

[88] Zhou J.G., Herscovici D., Chen C.C. (2000). Parametric process optimization to improve the accuracy of rapid prototyped stereolithography parts. Int. J. Mach. Tools Manuf..

[89] Arnold C., Monsees D., Hey J., Schweyen R. (2019). Surface quality of 3D-printed models as a function of various printing parameters. Materials.

[90] Sitek P.W.-M.R., Bolek J.Z., Mizera J., Kurzydlowski K.J. (2015). A Study on Technological Properties of Investment Casting Waxes. Adv. Appl. Plasma Sci..

[91] Chantarapanich N., Puttawibul P., Sitthiseripratip K., Sucharitpwatskul S., Chantaweroad S. (2013). Study of the mechanical properties of photo-cured epoxy resin fabricated by stereolithography process. Songklanakarin J. Sci. Technol.

[92] Olayan H.B., Hami H.S., Owen E.D. (1996). Photochemical and thermal crosslinking of polymers. J. Macromol. Sci. Part C Polym. Rev..

[93] Bagheri A., Jin J. (2019). Photopolymerization in 3D Printing. ACS Appl. Polym. Maters..

[94] Decker C., Viet T.N.T., Decker D., Weber-Koehl E. (2001). UV-radiation curing of acrylate/epoxide systems. Polymer (Guildf).

[95] Wang F., Wang F. (2017). Liquid Resins-Based Additive Manufacturing. J. Mol. Eng. Mater..

[96] Bartolo P.J., Gaspar J. (2008). Metal filled resin for stereolithography metal part. CIRP Ann. Manuf. Technol..

[97] Kaczmarek H. (2013). Effect of Irgacure 651 Initiator on Poly(Methyl Methacrylate) Photostability Studied by UV-Vis Spectroscopy. Open Process Chem. J..

[98] Green W.A. (2010). Industrial Photoinitiators: A Technical Guide.

[99] A New Photoinitiator for Cationic Curing from Ciba Specialty Chemicals. https://www.chemeurope.com/en/news/10417/a-new-photoinitiator-for-cationic-curing-from-ciba-specialty-chemicals.html.

[100] Zhang J., Xiao P. (2018). 3D printing of photopolymers. Polym. Chem..

[101] Barner-Kowollik C., Bastmeyer M., Blasco E., Delaittre G., Müller P., Richter B., Wegener M. (2017). 3D laser micro-and nanoprinting: Challenges for chemistry. Angew. Chemie Int. Ed..

[102] Weng Z., Zhou Y., Lin W., Senthil T., Wu L. (2016). Structure-property relationship of nano enhanced stereolithography resin for desktop SLA 3D printer. Compos. Part A Appl. Sci. Manuf..

[103] Malshe H., Nagarajan H., Pan Y., Haapala K. (2015). Profile of Sustainability in Additive Manufacturing and Environmental Assessment of a Novel Stereolithography Process. International Manufacturing Science and Engineering Conference.

[104] Brady G.A., Chu T.-M., Halloran J.W. Curing behavior of Ceramic Resin for Stereolithography. Proceedings of the 1996 International Solid Freeform Fabrication Symposium.

[105] Chartier T., Chaput C., Doreau F., Loiseau M. (2002). Stereolithography of structural complex ceramic parts. J. Mater. Sci..

[106] Zhou W., Li D., Wang H. (2010). A novel aqueous ceramic suspension for ceramic stereolithography. Rapid Prototyp. J..

[107] Griffith M.L., Halloran J.W. (2005). Freeform Fabrication of Ceramics via Stereolithography. J. Am. Ceram. Soc..

[108] Corcione C.E., Greco A., Montagna F., Licciulli A., Maffezzoli A. (2005). Silica moulds built by stereolithography. J. Mater. Sci..

[109] Bian W., Li D., Lian Q., Zhang W., Zhu L., Li X., Jin Z. (2011). Design and fabrication of a novel porous implant with pre-set channels based on ceramic stereolithography for vascular implantation. Biofabrication.

[110] Zhu J.-H., Li Q., Zhang W.-H. (2014). The periodic resin configuration design for ceramic-resin composite structure using topology optimization. Int. J. Simul. Multidiscip. Des. Optim..

[111] Hague R., Dickens P.M. Requirements for the Successful Autoclaving of Stereolithography Models in the Investment Casting Process. Proceedings of the Second National Conference on Rapid Prototyping and Tooling Research.

[112] Hague R., Dickens P.M. (2001). Improvements in investment casting with stereolithography patterns. Proc. Inst. Mech. Eng. Part B J. Eng. Manuf..

[113] Wang D., Dong A., Zhu G., Shu D., Sun J., Li F., Sun B. (2019). Rapid casting of complex impeller based on 3D printing wax pattern and simulation optimization. Int. J. Adv. Manuf. Technol..

[114] Li H., Chandrashekhara K., Komaragiri S., Lekakh S.N., Richards V.L. (2014). Crack prediction using nonlinear finite element analysis during pattern removal in investment casting process. J. Mater. Process. Technol..

[115] Yao W.L., Leu M.C. (1999). Analysis of shell cracking in investment casting with laser stereolithography patterns. Rapid Prototyp. J..

[116] Zhao H., Xu M., Li H., Everhart W., Lekakh S., Richards V., Chandrashekhara K., Nam P. Characterization of Low Density Polymer Patterns for Large Steel Investment Casting. Proceedings of the Investment Casting Institute 58th Technical Conference & Equipment Expo.

[117] Li Q., Zhu J.H., Zhang Y.H., Zhang W.H. (2013). The topology optimization of ceramic-resin composite structure under thermal and mechanical loads. Appl. Mech. Mater..

[118] Komaragiri S., Li H., Lekakh S., Chandrashekhara K., Richards V. Effects of complex geometry, shell thickness and firing regimes on shell cracking in industrial investment casting shells during rigid polymer pattern removal. Proceedings of the CastExpo and the Metalcasting Congress.

[119] Chen X., Li D., Wu H., Tang Y., Zhao L. (2011). Analysis of ceramic shell cracking in stereolithography-based rapid casting of turbine blade. Int. J. Adv. Manuf. Technol..

[120] Harun Z., Kamarudin N.H., Ibrahim M., Idris M.I., Ahmad S. (2015). Reinforced green ceramic shell mould for investment casting process. Advanced Materials Research.

[121] Lu Z.L., Fan Y.X., Miao K., Jing H., Li D.C. (2014). Effects of adding aluminum oxide or zirconium oxide fibers on ceramic molds for casting hollow turbine blades. Int. J. Adv. Manuf. Technol..

[122] Jones S., Yuan C. (2003). Advances in shell moulding for investment casting. J. Mater. Process. Technol..

[123] Dougherty T.K., Elias W.E., Thelander T.C., Bhavnani M.N. (1995). Resin Composition and Process for Investment Casting Using Stereolithography. U.S. Patent.

[124] Murphy E.J., Ansel R.E., Krajewski J.J. (1989). Investment Casting Utilizing Patterns Produced by Stereolithography. U.S. Patent.

